# How Well Do Randomized Trials Inform Decision Making: Systematic Review Using Comparative Effectiveness Research Measures on Acupuncture for Back Pain

**DOI:** 10.1371/journal.pone.0032399

**Published:** 2012-02-28

**Authors:** Claudia M. Witt, Eric Manheimer, Richard Hammerschlag, Rainer Lüdtke, Lixing Lao, Sean R. Tunis, Brian M. Berman

**Affiliations:** 1 University of Maryland School of Medicine, Center for Integrative Medicine, Baltimore, Maryland, United States of America; 2 Charité University Medical Center, Institute for Social Medicine, Epidemiology and Health Economics, Berlin, Germany; 3 Research Department, Oregon College of Oriental Medicine, Portland, Oregon, United States of America; 4 Carstens Foundation, Essen, Germany; 5 Center for Medical Technology Policy, Baltimore, Maryland, United States of America; University of Louisville, United States of America

## Abstract

**Background:**

For Comparative Effectiveness Research (CER) there is a need to develop scales for appraisal of available clinical research. Aims were to 1) test the feasibility of applying the pragmatic-explanatory continuum indicator summary tool and the six CER defining characteristics of the Institute of Medicine to RCTs of acupuncture for treatment of low back pain, and 2) evaluate the extent to which the evidence from these RCTs is relevant to clinical and health policy decision making.

**Methods:**

We searched Medline, the AcuTrials™ Database to February 2011 and reference lists and included full-report randomized trials in English that compared needle acupuncture with a conventional treatment in adults with non-specific acute and/or chronic low back pain and restricted to those with ≥30 patients in the acupuncture group. Papers were evaluated by 5 raters.

**Principal Findings:**

From 119 abstracts, 44 full-text publications were screened and 10 trials (4,901 patients) were evaluated. Due to missing information and initial difficulties in operationalizing the scoring items, the first scoring revealed inter-rater and inter-item variance (intraclass correlations 0.02–0.60), which improved after consensus discussions to 0.20–1.00. The 10 trials were found to cover the efficacy-effectiveness continuum; those with more flexible acupuncture and no placebo control scored closer to effectiveness.

**Conclusion:**

Both instruments proved useful, but need further development. In addition, CONSORT guidelines for reporting pragmatic trials should be expanded. Most studies in this review already reflect the movement towards CER and similar approaches can be taken to evaluate comparative effectiveness relevance of RCTs for other treatments.

## Introduction

Comparative Effectiveness Research (CER) has considerable potential to help health care providers as well as patients and clinicians to choose among currently available therapeutic options. Different definitions for CER have been published. In this paper we use the working definition as established by the Institute of Medicine (IOM) Committee, which defines CER as “the generation and synthesis of evidence that compares the benefits and harms of alternative methods to prevent, diagnose, treat, and monitor a clinical condition or to improve the delivery of care. The purpose of CER is to assist consumers, clinicians, purchasers, and policy makers to make informed decisions that will improve health care at both the individual and population levels” [1].

However, to date, the majority of clinical trials have assessed the efficacy of medical interventions rather than their effectiveness. To support more informed decision-making, there has been a call for more evidence on real world effectiveness from CER [2]. Available systematic reviews generally do not assess available evidence from a CER perspective – in other words, to examine the extent to which published trials are relevant to clinical and health policy decision making. On the contrary, appraisal of internal validity plays one of the most prominent roles in systematic reviews. For example, Cochrane reviews provide systematic information about possible bias within each study, but do not provide systematic information about the relevance of the study results for clinical and health policy decision-making.

For a better understanding of CER, it is essential to distinguish between ‘efficacy’ and ‘effectiveness’. ‘Efficacy’ refers to “the extent to which a specific intervention is beneficial under ideal conditions” [3]. Many randomized controlled trials are efficacy trials, particularly those conducted for regulatory drug approval. They aim to produce the expected result for an intervention under carefully controlled conditions chosen to maximize the likelihood of observing an effect if it exists. The trial population and setting of efficacy trials can differ in important ways from the clinical settings in which the interventions are likely to be used [4]. By contrast, ‘effectiveness’ is a measure of the extent to which an intervention, when deployed in the field in routine circumstances, does what it is intended to do for a specific population [3], and therefore can often be more relevant to policy evaluation and the health care decisions of providers and patients.

For randomized trials, the distinction between explanatory and pragmatic randomized trials was introduced in the 1960 s by Schwarz and Lelloch [5] and is also used in the CONSORT extension [6], another milestone publication on practical trials [7] and the pragmatic-explanatory continuum indicator summary (PRECIS) [8]. However, the term ‘explanatory’ can be misleading since pragmatic trials can also use an explanatory (confirmatory) statistical approach. Because of this potential confusion, we will use the terms ‘efficacy’ and ‘effectiveness’ for labeling the ends of this continuum. It is important to note that there is no sharp distinction between efficacy and effectiveness trials. Rather these terms exist in a continuum and the site along this continuum may differ for different features of the trial design.

This is reflected in the PRECIS tool [8] that was primarily developed to guide the design of RCTs along 10 dimensions of the efficacy-effectiveness continuum. In addition, the IOM has described six characteristics of CER (see Table 1) [1]. Both sets of criteria share the intent of describing the features of research that help inform clinical and health policy decisions. Use of these tools to assess existing trials may offer insights about the specific ways in which existing research has fallen short, and provide specific ideas about how to improve the quality and relevance of future trials. It is of major interest whether the available research can inform stakeholders. Do the existing criteria that define ‘pragmatism’ and CER that were developed for planning trials that inform clinical decision could be applied to the published trials as a means of evaluating and strengthening the evidence base for CER? Licensing drug trials usually have their main focus on efficacy, using placebo controls and objective outcome measures whenever possible. Because of these regulatory aspects, non-pharmacological studies would serve as better examples to show the whole range of an existing efficacy-effectiveness continuum.

**Table 1:** Rating details using the PRECIS criteria and the IOM characteristics.

criteria	Rating# max. diff.points	Intraclasscorrela-tion before/after	operationa-lization* good/moderate/difficult	comment		suggestions
**PRECIS criteria**						
1) eligibility criteria	1	−.12/.59	moderate	raters need good medical knowledge about the range of patients with this diagnosis in usual care	treatment guidelines could be used to aid decision making	
2) treatment flexibility intervention group	0	.82/1.00	good	usual care situation differs in countries and even US States, number of treatment always limited in interventional trials	more details in CONSORT guidelines	
3) practitioner expertise intervention group	1	.10/.69	moderate	expertise range differs between countries and even US States, often no data about usual care setting and limited information about selection procedure	more details in CONSORT guidelines	
4) treatment flexibility control group	1	.58/.95	moderate	publications often don't provide enough information about co-interventions, number of treatment always limited in interventional trials	more details in CONSORT guidelines	
5) practitioner expertise control group	1	.60/.92	moderate	publications don't provide enough information, expertise range differs between countries and even US States, often no data about usual care setting and limited information about selection procedure	more details in CONSORT guidelines	
6) follow up intensity	1	.02/.36	difficult	trial situation always differs from usual care, influence of telephone interviews, or questionnaires is difficult to operationalize	clear operationalization needed	
7) outcomes	1	−.20/−.20	difficult	raters need good knowledge about valid outcomes for the diagnosis, usual care situation on one end of the scale with no interference was difficult	more diagnoses specific standards e.g. in treatment guidelines needed	
8) patients' compliance	2	.28/.62	difficult	publications don't provide enough information	could be included in CONSORT guidelines	
9) practitioners' protocol adherence	1	.29/.68	difficult	publications don't provide enough information	could be included in CONSORT guidelines	
10) primary analysis	1	−.12/.77	good	older publications do not provide this information systematic, most trials do ITT and the relevant topic of subgroup analyses is missing in PRECIS	aspect of subgroup analysis should be included (see IOM)	
**IOM criteria**						
1) directly informing a specific clinical decision from the patient perspective or a health policy decision from the population perspective	3	−.17/.03	moderate	depends on health system, interpreted differently from different perspectives		
2) comparing at least two alternative interventions, each with the potential to be “best practice	2	−.09/.24	moderate	raters need good medical knowledge about treatments options and standards, treatment standards differ between countries, alternatives could be whole treatment packages and also usual care		treatment guidelines could be used to aid decision making
3) describing results at the population and subgroup levels	0	−.21/1.00	moderate	publications provide often none only partial results (e.g. p value for effect modification), items can be easily clearer operationalized		Data on effect modification, but also results for subgroups needed, should be included in CONSORT guidelines
4) measuring outcomes—both benefits and harms—that are important to patients	2	−.19/1.00	moderate	raters need good knowledge about valid outcomes for the diagnosis, difficult to decide which emphasis outcome and safety has in the rating		more diagnoses specific standards e.g. in treatment guideline needed that could linked
5) employing methods and data sources appropriate for the decision of interest	1	−.03/.03	moderate	publications don't provide enough information about the rational and setting for trial question		
6) conducted in settings that are similar to those in which the intervention will be used in practice.	2	.37/.69	moderate	publications don't provide enough information about usual setting for the intervention, setting differs between countries		more details in CONSORT guidelines

# after consensus max difference of points (scale 1–5, 1 = max. efficacy to 5 = max. effectiveness) for each of the trials for this criteria,

* qualitative result from the discussion within the consensus procedure.

CER is especially valuable for those disorders that are the most common and most costly to society, have the highest morbidity rates, and a great degree of variation in their practice [9]. Low back pain has a high lifetime prevalence, is one of the most common reasons for visits to a physician [10] and results in high health care expenses [11]. An estimated 8 million Americans have used acupuncture as a treatment for persistent disabling pain conditions that include chronic low back pain [12], and clinical relevance of acupuncture for chronic low back pain in usual care is highlighted by a recent clinical expertise paper on acupuncture for chronic low back pain in the New England Journal of Medicine [13]. In this paper, we explore the efficacy/effectiveness continuum in the context of RCTs that assess the impact of acupuncture on low back pain.

This systematic review aims to 1) test the feasibility of applying the PRECIS tool and the IOM CER characteristics to RCTs of acupuncture for treatment of low back pain, and 2) evaluate the extent to which the evidence from these RCTs is relevant to clinical and health policy decision making.

## Methods

### Data sources and searches

We identified trials using the following search strategy:

- AcuTrials™ Database [14] Feb 10, 2011 searched for low back pain and a comparator group, which was standard care/usual care or no treatment. This database was created by the Research Department, Oregon College of Oriental Medicine, Portland, OR as a comprehensive database that includes all RCTs and systematic reviews on acupuncture published in English.
- Medline 1966 to Feb 17, 2011 searched for ‘back pain and acupuncture’ or ‘back pain and Chinese Medicine’ or ‘back pain and Traditional Chinese Medicine’ using the limits Clinical Trial, Meta-Analysis, Randomized Controlled Trial, English.
- Hand-searching for applicable trials, including the two most recent meta-analyses [15], [16].

### Study selection

#### Types of trials

We included controlled trials in which allocation to treatment was explicitly randomized. Trials were excluded that used an inappropriate method of randomization, e.g. open alternation or lottery.

#### Types of participants

Trials conducted among adult patients suffering from non-specific acute and/or chronic low back pain were included. Trials including patients with specific low back pain, e.g., sciatica or pelvic and lumbar pain during pregnancy, were excluded.

#### Types of interventions

The treatments considered had to at least involve needle insertion at acupuncture points, pain points or trigger points, and be described as acupuncture. The control interventions considered were conventional treatments (drugs, relaxation, physical therapies, self care etc.). Trials with additional acupuncture interventions based on usual care or other conventional interventions were included. Trials in which patients in the control group had no treatment or only rescue medication or TENS were excluded because they were not considered adequate conventional treatment interventions.

#### Types of publications

We included only English-language full papers that reported results of single trials. Follow-up publications, protocol publications, diagnostic trials, publications on intervention details, and publications that reported only economic results were excluded.

#### Sample size

Because we were mainly interested in the efficacy-effectiveness continuum and due to higher variance it is difficult to assess effectiveness with very small samples, we predefined arbitrary to include only those RCTs with ≥30 patients in the acupuncture group.

### Data Extraction and Quality Assessment

**Figure 1:**
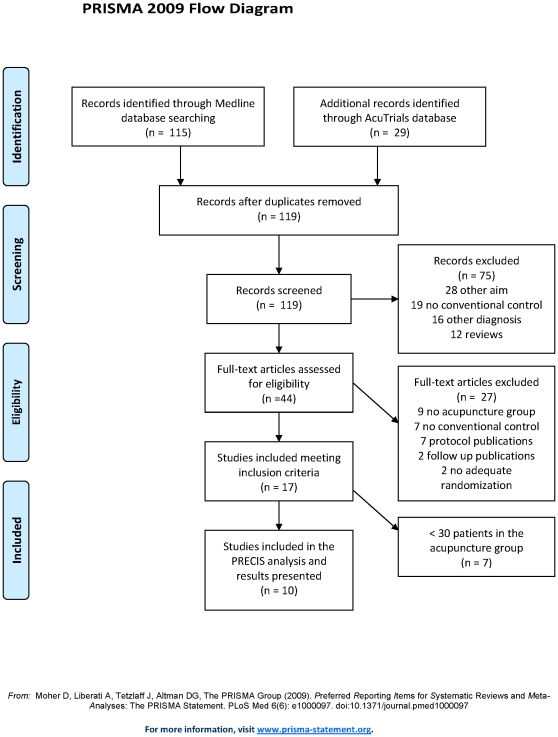
Study selection.

Selection of trials and preliminary data extraction were performed by one rater (CMW). As a first step, references retrieved from Medline and the AcuTrials database were combined and duplicates were removed. All remaining abstracts were screened and trials that were clearly irrelevant were excluded (e.g., specific low back pain, only sham control or no control group, see Figure 1 for details). In addition, reference lists of recent systematic reviews [15], [16] were checked, but did not reveal further unique trials. For the abstracts meeting inclusion criteria, the full papers were obtained and were formally re-checked to exclude ineligible papers. Information on methods, patients, interventions, outcomes and results was extracted from the included trials and entered into an Excel spreadsheet. Special attention was given to sample size, details and rationale of the intervention and comparator groups, the terminology used (efficacy or effectiveness), the test hypothesis (non-inferiority or superiority) and the effect size. If the effect size was not given in the original publications, it was extracted from published meta-analysis.

### Data syntheses and analyses

The protocol of the systematic review was predefined. For all included trials, the efficacy-effectiveness continuum was assessed using both the ten PRECIS criteria [8] and the six Institute of Medicine (IOM) defining characteristics of CER [17] To allow a clearer approach, we converted the terminology from ‘explanatory/pragmatic’ to ‘efficacy/effectiveness.’ Assessment of trials (Table 2) was performed independently by 5 raters using an enhanced quantified version of the PRECIS and IOM characteristics with a scale of 1–5 for each criterion (1 = maximal efficacy to 5 = maximal effectiveness). This allowed calculation of inter-rater correlations and to present results in figures. The five raters came from different backgrounds (MD and PhD), each had more than 10 years of experience in clinical research, had worked on aspects of research methodology, and had experience in systematic reviews and acupuncture trials. Rating was done independently, results were sent from each rater to CMW, and RL performed the statistics. For the final results, each item was discussed in a conference call between all raters until a consensus was reached.

**Table 2:** Trials on non specific low back pain with a conventional treatment comparator and>30 patients in the acupuncture group.

Author	N Acu/Con	Result SMD	Acupunc- ture#	Acu rational	Setting	Comparator details	Cointervention	Ad on	comparator details presented	Rating PRECIS criteria (5-effectiveness/1-efficacy)										Rating IOM criteria (5–1)					
**Acute non specific low back pain**										1	2	3	4	5	6	7	8	9	10	1	2	3	4	5	6
**Eisenberg 2007 (19)**	58/150	na	free, max 10 sessions	as usual	physicians, practitioners outpatient practices	treatment follows hospital guideline care (NSAID, muscle relaxants, education, activity alteration)	treatment follows hospital guideline care (NSAID, muscle relaxants, education, activity alteration)	yes	number of visits	3.4	4.0	3.6	4.0	3.6	2.4	4.2	4.8	5.0	4.0	4.6	4.6	2.0	4.8	4.8	4.2
**Chronic non specific low back pain**																									
**Witt 2006 (26)**	1451/1390	0.43* (0.38;0.49)	needle, free, max 15 sessions	as usual	physicians outpatient practices	patients were free to seek care in the health insurance system	usual care	yes	pain medication in both groups reported	4.4	5.0	4.0	5.0	5.0	3.6	4.4	4.4	5.0	4.0	4.6	4.0	2.8	4.6	4.8	4.0
**Haake 2007 (21)**	387/387	0.56* (0.43;0.70)	needle, semi-standard, 10 or 15 session	consen-sus	physicians outpatient practices	German guideline based treatment (physiotherapy, exercise, NSAID)	rescue medication	no	conventional group type and frequency of treatment	3.0	2.0	3.0	4.0	3.8	2.8	4.4	4.2	3.2	4.0	4.4	4.6	1.0	4.2	4.8	4.0
**Thomas 2006 (27)**	160/81	0.34* (0.03;0.65)	needle, free, max 10 sessions	as usual	practitioners outpatient practices	treatment as provided by their GPs	treatment as provided by their GPs	yes	both groups type of treatment	4.4	5.0	3.8	5.0	5.0	3.6	4.4	4.6	5.0	3.4	4.4	4.4	2.0	4.6	4.8	4.0
**Cherkin 2009 (20)**	157/161	na	needle, free, 10 sessions	by one diagnos-tician	2 research clinics, outpatients	treatment as provided by their physicians	self care book, usual care	yes	% of patients with physician and practitioner visits	3.4	3.0	3.0	5.0	5.0	2.8	4.4	4.2	4.2	3.4	4.4	4.2	1.0	4.4	4.8	3.0
**Cherkin 2001 (22)**	94/90	0.24* (0.00;0.48)	needle +E-stim, max 10 sessions	consen-sus	practitioners outpatient practices	self care materials (book, videotapes)	usual care	yes	treatments both groups; % patients with as non study visits	3.0	5.0	3.4	2.0	na	2.8	4.4	4.4	4.2	4.0	4.4	3.4	2.0	4.6	4.6	4.0
**Molsberger 2002 (23)**	65/60	0.62** (0.26;0.97)	needle, standard, 12 sessions	literature	rehabilitation clinic, inpatients	physiotherapy, exercise, education, mud packs, IR-heat, diclofenac (3×50 mg on demand)	physiotherapy, exercise, education, mud packs, IR-heat, diclofenac (3×50 mg on demand)	yes	not presented	2.6	3.0	2.6	2.0	3.0	3.4	4.4	4.9	na	3.2	4.4	4.4	1.0	4.2	4.6	3.0
**Leibing 2002 (24)**	40/46	0.86** (0.42;1.31)	needle, standard, 20 sessions	literature	university hospital outpatient cinic	physiotherapy	physiotherapy	yes	not presented	3.4	1.0	2.0	2.4	2.2	3.2	4.4	4.8	na	3.2	4.0	4.4	1.0	4.2	4.8	2.8
**Szczurko 2007 (29)**	39/30	na	needle, standard, 24 sessions	unclear	practitioners on site at a plant	self care booklet and physiotherapy advice and relaxation techniques		no	percentage of patients compliant to dietry advice and to exercise	3.4	1.0	2.8	2.4	2.4	3.0	4.4	2.4	4.2	5.0	3.6	3.8	1.0	4.0	4.2	3.2
**Meng 2003 (30)**	31/24	0.50** (0.08;1.09)	needle, standard, 10 sessions	unclear	aneastesio-logists at hospital, outpatients	treatment as before provided by their physicians (NSAID, aspirin, non narcotic analgesics)	treatment as before provided by their physicians (NSAID, aspirin, non narcotic analgesics)	yes	patients with medication and use of other CAM treatments	2.6	2.0	2.8	3.4	5.0	3.0	4.6	3.8	na	3.0	4.0	4.6	3.0	4.2	4.6	3.2

Acu = acupuncture, Con = control, na = not available,

* primary endpoint from individual patient data meta-analysis (Vickers Trials 2010),

** short term effect on pain scales from recent meta-analysis (Yuan Spine 2008),

# standard = standardized treatment protocol, free = acupuncture as usual PRECIS: 1) Eligibility criteria, 2) Flexibility acu, 3) Practitioner expertise acu, 4) Flexibility control, 5) Practitioner Expertise control, 6) follow up, 7) Outcomes, 8)Patient compliance, 9) Practitioner adherence, 10) primary analysis IOM: 1) Informing decision making, 2) Comparing at least two alternatives, 3) Results for population and subgroups, 4) Patient relevant outcomes incl. safety, 5) Appropriate methods, 6) Setting close to reality.

Agreements between raters (inter-rater reliability) were calculated separately for each item and each time point (before and after the consensus conference) by intraclass-correlations as defined by Shrout and Fleiss [18].

## Results

### Search Results

Altogether, 119 abstracts were identified: 115 from Medline and 4 additional from the AcuTrials™ database; no further unique abstracts were identified from the recent systematic reviews. Of these abstracts, 44 full papers were screened, and 10 trials, including 4901 total patients (2482 acupuncture and 2419 control) met the eligibility criteria and were subjected to data extraction (see Figure 1).

### Included trials

One trial focused on acute low back pain [19], while all the others were on chronic pain low back pain. One trial included two acupuncture groups: a standardized group and an individualized acupuncture group [20]. For this analysis, we used the individualized acupuncture group because we assumed this group to be closer to usual care. Within the 10 trials, four included a sham acupuncture group [21]–[24] and four included an economic analysis [22], [25]–[27]. Only two trials used a complex intervention. In the trial by Cherkin [28], other Chinese medicine interventions such as cupping and moxibustion, were allowed. However, in the trial by Szczurko [29], acupuncture was delivered within a naturopathic treatment, which included exercise and dietary advice. All trials tested for superiority of acupuncture treatment. None of the trials aimed to evaluate the non-inferiority of acupuncture compared to conventional care. All ten trials were published in peer reviewed medical journals with relevant impact (Arch Int Med, BMJ, Am J Epi, Pain, PLOS One, Rheumatology, Spine).

### Interrater Reliability of Ratings

Raters judged the general difficulty of applying the criteria on a scale from 0–10 (0 = very easy; 10 = very difficult) as 6 (median; range 2–7) for PRECIS and 8 (median; range 6–10) for the IOM criteria. The first independent ratings of the efficacy-effectiveness continuum were highly heterogeneous between trials and between raters. This resulted in low inter-rater reliability estimates (Table 2). Missing information in the publications and difficulties in operationalizing the criteria were cited most frequently as the main reasons for the high rater variation in initial scoring of the trials (Table 1). Improved inter-rater reliability was found after the consensus discussion. The consensus process benefitted from each rater's experience in conducting and/or assessing trials on low back pain and acupuncture. Although there was still no full consensus between raters, the maximum difference was 2 points.

### Mean Ratings of the Efficacy – Effectiveness Continuum

**Figure 2:**
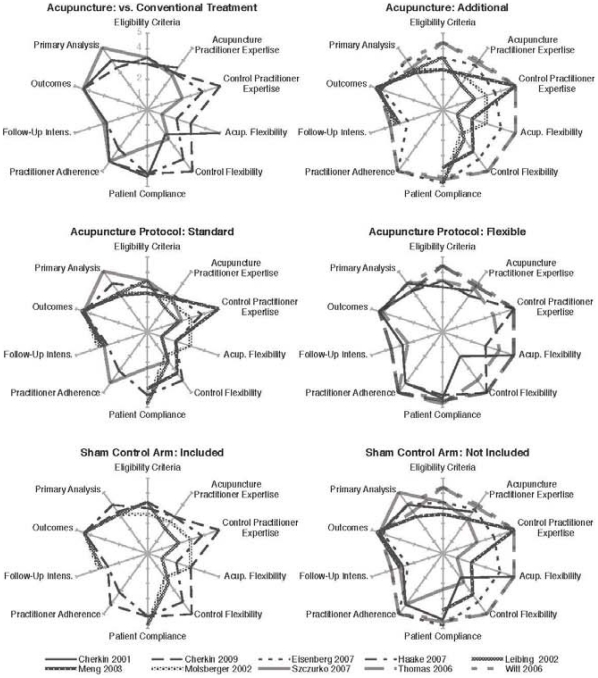
PRECIS scoring for the 10 included trials comparing different methodological aspects (second rating after consensus procedure), a larger rounder figure would correlate with a higher score on PRECIS representing more the effectiveness side.

Details on the trials are presented in Table 2. The trials by Thomas et al [27] and Witt et al [26] that compared adjunctive acupuncture to usual care alone had high effectiveness scores on the efficacy-effectiveness continuum and could serve as examples for trials that aim to represent a usual care situation, whereas those trials which included an additional sham control arm [20], [21], [23], [24] had higher efficacy scores representing a more experimental approach. This corresponded to the wording in the papers: Only those trials that included a sham control arm used the term ‘efficacy;’ all other trials used the term ‘effectiveness’. Interestingly, most trials that scored higher on the efficacy side of the continuum were less standardized than usually observed in drug research. The results showed that, for each trial, the placement along the efficacy-effectiveness continuum is multi-dimensional and varied for the different criteria within a given trial (Figure 2). Overall, when evaluating acupuncture as an adjunctive treatment that allowed more flexible treatment protocols, trials had higher effectiveness scores than trials that evaluated acupuncture as a treatment alternative and used a more standardized treatment protocol (Figure 2).

An interesting exploratory observation is that those trials that reported more narrow eligibility criteria and a more standardized acupuncture intervention [23], [24], [30] resulted in larger effect sizes (≥0.5, Table 2) than trials that reported a more heterogeneous patient sample and a flexible acupuncture treatment (effect size≤0.5, Table 2) [26], [27].

## Discussion

Using available criteria for planning CER to evaluate the efficacy-effectiveness continuum of published trials resulted in large heterogeneity between raters and items, which was partly solved by a consensus procedure. This was mainly due to information missing from the publications and to difficulties in operationalizing the criteria. Our focus on RCTs assessing acupuncture for low back pain allowed the inclusion of a number of high quality trials representing a broad spectrum of clinical research in the efficacy-effectiveness continuum. Trials that have a more flexible acupuncture treatment protocol and no further placebo control arm scored closer to effectiveness.

This is a systematic analysis that has tested the feasibility of appraising the efficacy-effectiveness continuum of randomized controlled trials. Advantages of the systematic review include its innovative scope on the process of appraisal, high quality studies covering the efficacy-effectiveness continuum, and that the scoring was done by 5 independent raters using two different sets of criteria. The review process benefitted from the experience of the selected raters in the design, performance and/or assessment of the field of research. Discussions between raters improved the inter-rater reliability significantly. This underlines the complex aspects of the efficacy-effectiveness continuum and the need for rater training. Limitations were that only one rater selected the papers, that secondary papers (e.g., on treatment details) were not included, and that randomized trials are only one part of CER and do not represent the whole spectrum of evidence. However, Cochrane reviews, which are often used to assist in decision-making, also focus on RCTs and primarily concentrate on the main paper presenting the results. Another limitation is that both criteria lists (PRECIS and IOM) were developed to guide new trials and not to assess published trials. However, the present study provides insights into the advantages and limitation of single items and indicates that, following the definition and main characteristics of CER, the ten PRECIS criteria and six IOM characteristics seem plausible candidates for the evaluation of existing research and could form a basis for a future evaluation instrument. That the items of the PRECIS tool have relevance for appraising published studies is supported by the very recent review by Koppenaal et al [31]. The authors used the PRECIS tool on two meta-analyses, scored the single items, and came to the conclusion that PRECIS can provide useful estimates on how single studies and the whole review are placed within the efficacy effectiveness continuum. Interestingly the authors used a similar scale from 1 to 5. However, they did either provide information on inter-rater variability nor details on advantages and limitations of single PRECIS items which can inform its further development.

The origin of some of the effect sizes presented in this review could be seen as a limitation. It was not the aim of this review to perform a meta-analysis and because of this effect sizes were taken from the literature and only used as an exploratory aspect for orientation.

The present findings reveal that the place of a trial in the efficacy-effectiveness continuum is multidimensional, indicating it is even more complicated to unambiguously label a trial as efficacy or effectiveness. From the scoring of the trials, it is clear that two of the RCTs [26], [27] were designed mainly as effectiveness trials, whereas others were designed more as efficacy trials [23], [24], [29]. Interestingly, two of the trials [20], [21], both including a sham control, standardized their acupuncture intervention much more than their conventional treatment control. None of the trials included all available patients, but eligibility criteria varied from relatively narrow to relatively wide.

In the early 1970's, when Asian medicine including acupuncture began its most recent migration to the West, researchers adopted the randomized controlled trial to investigate acupuncture without knowing Asian medicine had a long history [32]. Because of this evidence from those trials was often rejected as invalid and was therefore ignored. The discussion and demand for evidence that is generated in a way that satisfies decision-making started early [33] and most studies in this review already reflect the movement toward an evidence base that can inform decisions makers. Acupuncture for low back pain can serve as a good example for different options of randomized studies within CER. On one hand, both large studies that evaluated acupuncture as adjunct to usual care represent a unique way that RCTs can more closely reflect the reality of a usual care setting [26], [27]. On the other hand, those trials that had both a standard care/usual care control and a sham control arm, but still tried to keep their acupuncture intervention more flexible are good examples for a middle ground in the efficacy-effectiveness continuum [21]. Overall, the last decade of the acupuncture studies on low back pain provides useful information for the design of future randomized trials in other fields of non-pharmacological research.

In the scoring process of the trials appraising the eligibility criteria was not always easy. Therefore, it would be useful to analyze heterogeneity in addition to get better knowledge about the population in the studies [34]. It is important that trials with more heterogeneous populations result in higher outcome variances and smaller effect sizes, which must be taken into account when planning the sample sizes for future trials assessing CER.

Furthermore, CER is susceptible to systematic error [35]. The attempt to achieve methodological purity can result in clinically meaningless results, while attempting to achieve full generalizability can result in invalid and unreliable results. Achieving a creative tension between the two is crucial [36] and the relevance of the results has to be put into accordance with the rigor of the results. In CER, the evaluation of effect modifications and stratifications play a crucial role [37] to allow for conclusions on specific subgroups. This is one of the IOM criteria, but was not represented in the PRECIS score. Although the trials in our analysis were mainly published in high-ranking journals, none of the trials that scored more on the effectiveness side of the continuum gave detailed information about subgroups. For decision-making, this aspect should be strengthened in future trials and should be included in the criteria list for evaluation of the efficacy-effectiveness continuum.

One problem that came up during the rater consensus procedure was the information missing from the main publications. It is highly recommended to include in future review processes also all available secondary papers. However, in the case of the included studies information on selection procedure of practitioners, as well as for patient compliance measures and practitioner adherence to protocol would not have been complete. In addition, it would be helpful to know more about the setting in which the treatment is typically carried out in each respective country and how much the trial setting differs from the clinical treatment setting. Although standards for reporting clinical trials (CONSORT [6], STRICTA [38]) mention the most relevant aspects, the above mentioned aspects, such as describing the usual setting for this treatment in detail and providing clear information on patients' compliance and practitioner adherence, are not adequately represented in the CONSORT guidelines and should be discussed in future revisions.

### Conclusion

It is of high relevance for stakeholders to appraise the extent to which published trials are relevant to clinical and health policy decision-making. A systematic instrument, which can be also used in systematic reviews, needs further development. The available instruments for planning randomized studies for CER could provide a basis for this, but would need further development that includes more defined operational criteria and a rater's training manual. In addition, CONSORT guidelines for reporting RCTs should be more extended, fostering on reporting more details on CER relevant aspects. Most studies in this review already reflect the movement toward an evidence base that can inform both decision-makers and provide useful information for the design of randomized trials for other non-pharmacological treatments.
